# Microplastic exposure and human health risks across the life cycle: a focus on reproduction, development, and aging

**DOI:** 10.3389/fcell.2026.1778576

**Published:** 2026-03-13

**Authors:** Guosheng Liu, Tian Shi, Shengyao Tang, Xia Huang, Xiao Liu

**Affiliations:** 1 Hubei Provincial Key Laboratory of Occurrence and Intervention of Rheumatic Diseases, Enshi, Hubei, China; 2 Department of Medical Laboratory, Minda Hospital of Hubei Minzu University, Enshi, Hubei, China

**Keywords:** child development, emerging pollutants, human fertility, microplastic pollution, mitochondrial dysfunction, pregnancy

## Abstract

Microplastic pollution has emerged as a critical global environmental and public health challenge. These small plastic particles of diverse origins are ubiquitously distributed in aquatic, atmospheric, terrestrial, and food systems, entering the human body through ingestion, inhalation, and dermal contact, thereby creating complex lifelong exposure scenarios. Accumulating evidence indicates that microplastics (MPs) not only pose threats to key early-life stages—including reproductive health, pregnancy maintenance, fetal development, and child growth—but may also systematically accelerate the aging process and increase the risk of age-related diseases. This review provides a comprehensive synthesis of the physicochemical properties, environmental distribution, human exposure pathways, and life-cycle health impacts of MPs. It elaborates on their specific adverse effects on the reproductive system and their interference with fetal and child development. Furthermore, it delves into the core molecular mechanisms by which MPs drive cellular and tissue aging, primarily through the induction of mitochondrial dysfunction, oxidative stress, and chronic inflammation. The review also summarizes current research limitations concerning methodological standardization and epidemiological evidence, while outlining priority areas for future investigation. By integrating evidence across the life course, this review aims to establish a solid theoretical foundation for understanding the composite health risks of MPs, identifying vulnerable life stages, and informing the development of scientific prevention and intervention strategies.

## 1 Introduction

Microplastic pollution has emerged as a defining global environmental and public health challenge. Driven by exponentially increasing plastic production and often inadequate waste management, plastic debris is now ubiquitous across ecosystems, from remote glacial regions to deep-sea sediments (Andrady, 2011; Singh and Mishra, 2023). Among these pollutants, MPs—typically defined as plastic particles less than 5 mm—are of particular concern due to their small size, persistence, and potential for widespread dispersal and bioaccumulation.

Their environmental prevalence and complex behavior, including acting as vectors for other contaminants and leaching inherent additives, firmly align MPs with the category of “emerging contaminants” (Sun et al., 2024). More critically, their micro-to nano-scale dimensions facilitate uptake by organisms through ingestion, inhalation, and dermal contact (Jambeck et al., 2015; Lin et al., 2023; Chen S et al., 2023). Growing evidence indicates that these particles can traverse significant biological barriers, such as the intestinal mucosa, blood-brain barrier, and even the placental barrier, raising profound concerns about their direct interaction with human tissues and cellular systems (Jambeck et al., 2015; Lin et al., 2023).

While considerable research has detailed the environmental occurrence and ecotoxicological effects of MPs, a crucial gap remains in systematically linking human exposure to specific and sensitive health endpoints across the life course. Early life stages—encompassing reproductive health, pregnancy, fetal development, and childhood growth—are potentially highly vulnerable to toxic insults. Furthermore, emerging hypotheses suggest that chronic MP exposure may contribute to accelerated aging and the pathogenesis of age-related diseases through fundamental cellular disruptions. However, the exact exposure-dose relationships, detailed molecular mechanisms, and compounded risks from co-exposure with other environmental stressors in humans are poorly understood, severely hampering evidence-based risk assessment and public health intervention (Yang Z et al., 2023).

**FIGURE 1:**
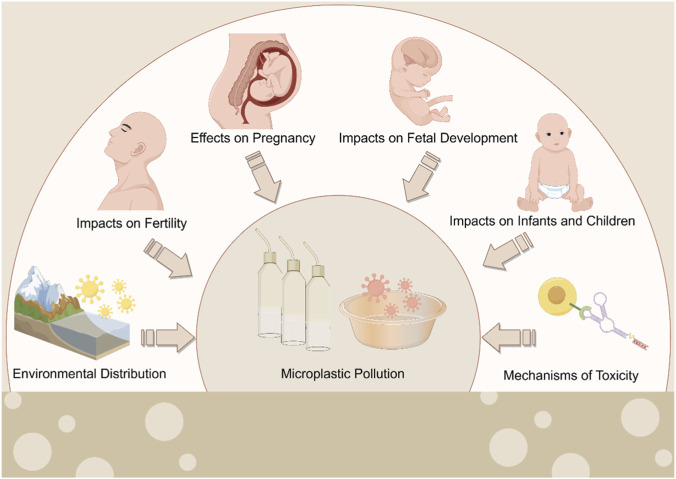
The impact of MPs on human fertility, pregnancy, and child development.

This review, therefore, aims to synthesize current knowledge on the lifecycle health impacts of MPs, with a focused analysis on their threats to reproduction, development, and aging processes. By integrating evidence on exposure pathways and toxicity mechanisms, we seek to clarify the composite health risks posed by MPs, identify critical windows of vulnerability, and provide a scientific foundation for future research and preventive strategies (Figure 1).

## 2 Microplastics in the environment and human exposure

### 2.1 Properties of microplastics

The physical and chemical properties of MPs collectively determine their transport, transformation in the environment, and potential ecological and health risks, forming the key basis for systematically assessing their environmental behavior and toxicological effects.

Regarding physical properties, size is one of the most important characteristics of MPs, typically defined as particles less than 5 mm, further categorized into micron-scale and nano-scale plastics (Galloway et al., 2017). Size directly influences their environmental fate and bioavailability; smaller particles are more easily ingested by organisms and may penetrate biological barriers. MPs exhibit diverse morphologies, including fibers, fragments, pellets, films, and microbeads (Wright and Kelly, 2017). Different shapes affect their mobility and adsorption behavior; for instance, fibers tend to accumulate in sediments, while spherical particles are more prone to resuspension. The color of MPs is also a significant attribute, potentially increasing the risk of mistaken ingestion by organisms. For example, brightly colored microplastic particles are easily mistaken for prey and consumed by aquatic organisms, showing significantly higher detection rates in fish gastrointestinal tracts compared to transparent particles (Li et al., 2023; Zhao et al., 2022). Density is a key parameter determining the behavior of MPs in aquatic environments; high-density plastics like PVC and PET tend to sink and accumulate in sediments, while low-density types like PE and PP float on the water surface (Kooi and Koelmans, 2019). This property directly affects the distribution of MPs in freshwater, marine, and terrestrial environments, thereby shaping exposure pathways for humans and wildlife. Furthermore, surface characteristics (e.g., roughness, porosity) and hydrophobicity significantly influence their interactions with other pollutants and biofilms (Catrouillet et al., 2021; Kooi and Koelmans, 2019; Xu et al., 2025). These physical attributes collectively regulate the environmental behavior of MPs, their capacity to carry pollutants, and their overall ecological and health impacts.

Regarding chemical properties, MPs are composed of various polymers, commonly including polyethylene, polypropylene, polystyrene, and polyvinyl chloride, among others. Different polymers possess varying chemical stability and resistance to degradation, directly affecting their persistence in the environment. For example, polyethylene and polypropylene exhibit high chemical inertness due to their saturated carbon chain structures, making them resistant to photodegradation and biodegradation in the natural environment, allowing them to persist for centuries (Gewert et al., 2015); thus, they are the most abundant polymer types in marine plastic accumulation zones. In contrast, while polystyrene has good chemical resistance, the benzene ring structure in its molecular chain is sensitive to UV light, making it prone to embrittlement and cracking under prolonged sunlight exposure, thus more easily fragmenting into secondary microplastic particles (Kubowicz and Booth, 2017; Pelegrini et al., 2019; Yang Z. et al., 2023). Beyond the inherent properties of the polymers, MPs may also release incorporated additives (e.g., plasticizers, stabilizers, flame retardants) and adsorb external pollutants from the environment; these processes collectively influence their toxicity and retention behavior in the environment (Zimmermann et al., 2019). Under environmental aging factors such as UV radiation, mechanical abrasion, and biological activity, MPs undergo surface oxidation, crack propagation, and the formation of functional groups, thereby altering their surface adsorption characteristics and biological interactions. Studies show that the adsorption capacity of UV-aged polypropylene MPs for copper ions can increase by up to 92 times (Han et al., 2021). Moreover, pollutants adsorbed onto MPs may desorb after entering organisms, enhancing their toxic effects. For instance, additives and monomers (e.g., phthalates, bisphenol A) released during degradation are known endocrine disruptors with potential health risks (Cao et al., 2022; Lo et al., 2021).

### 2.2 Environmental distribution

MPs, as persistent environmental pollutants, have been widely detected in various environmental media, including water bodies, the atmosphere, soil, and food systems. Characterized by environmental persistence, long-range transport potential, and bioaccumulation capacity, they pose potential risks to ecosystem function and public health that cannot be ignored.

In aquatic ecosystems, microplastic pollution is ubiquitous in water bodies such as rivers, lakes, groundwater, and oceans. Their sources are complex and diverse, encompassing both primary MPs directly input from urban runoff and industrial wastewater, and secondary MPs formed from larger plastic waste in the environment via physical fragmentation and photochemical degradation (Pastorino et al., 2025). Studies have confirmed that in marine environments, MPs are distributed from surface waters to abyssal plains, from coastal regions to the open ocean, and can undergo long-range transport via ocean currents, demonstrating significant environmental persistence. Notably, ocean gyres tend to form microplastic accumulation zones, the so-called “plastic vortices” (Cholewińska et al., 2025; Pastorino et al., 2025; Zhang et al., 2026). In freshwater systems, the distribution of MPs is significantly influenced by human activities, hydrodynamic conditions, and seasonal variations, often forming pollution hotspots downstream of urban areas, at wastewater treatment plant discharges, and in areas affected by agricultural runoff. Exceptionally high microplastic concentrations have been reported in waters downstream of major urban sewage inputs, near outlets of large wastewater treatment plants, and in lakes and reservoirs surrounding farmland reliant on plastic mulching and heavy application of sludge-based fertilizers (Blettler and Wantzen, 2019). Furthermore, MPs are not only present in water and sediments but can also be ingested by aquatic organisms, creating multi-pathway ecological and health exposure risks through trophic transfer and bioaccumulation.

Atmospheric microplastic pollution has become a global environmental issue, characterized by diverse sources and high mobility. Major sources include tire wear, shedding of synthetic fibers, resuspension of urban dust, and secondary release of MPs from terrestrial and aquatic environments (Allen et al., 2019; Xiao et al., 2023). The transport and deposition of atmospheric MPs are influenced by their physical properties (e.g., size, density), meteorological conditions, and human activities. Larger particles tend to deposit near emission sources, while smaller, less dense particles can remain suspended for long periods and undergo transboundary transport with air masses (Yang et al., 2021). Research shows that MPs have been detected in atmospheric deposition from urban and suburban areas to remote regions like high mountains and the polar regions, confirming their wide distribution range and complex transport pathways (Herzke et al., 2026). These inhalable particles can not only enter the human respiratory system causing potential toxic effects but also enter water and soil via dry and wet deposition, ultimately entering the food chain (Naggar et al., 2021; Lu et al., 2025). Moreover, due to their high specific surface area, MPs readily adsorb heavy metals, persistent organic pollutants, and microorganisms, acting as composite pollutant vectors that further exacerbate environmental and health risks.

Soil is a significant sink for MPs, posing potential threats to soil ecological functions and agricultural product safety. MPs enter the soil environment through various pathways including residual agricultural plastic film, application of sewage sludge and organic fertilizers, atmospheric deposition, and irrigation with contaminated water (Li et al., 2021; Munno et al., 2021; Adjama et al., 2024). These particles can alter soil structure and microbial activity, thereby affecting crop production and soil health. Current research has confirmed that microplastic pollution can inhibit seed germination rate and root development in cereal crops like wheat and rice (Du et al., 2024). The distribution of MPs in soil exhibits significant spatial heterogeneity, influenced by land use patterns, agricultural management practices, and soil properties (Koelmans et al., 2017). For instance, farmland soils with long-term use of plastic mulching and recycled water irrigation, as well as soils near urban and industrial areas, are more prone to high-level pollution. Additionally, the transport processes of MPs in soil are complex, involving vertical migration via bioturbation, root growth, and water movement, as well as horizontal dispersion via surface runoff and soil erosion. Of particular concern is that nano-scale plastic particles can be absorbed by plant roots and transported to edible parts, thereby entering the food chain and threatening agricultural product safety and human health (Allen et al., 2019; Itmec et al., 2025).

Microplastic contamination in food systems has become a significant issue for global food safety and public health. MPs enter the human food chain through various routes including agricultural products, aquatic products, and processed foods (Yadav et al., 2022). In agricultural production, MPs primarily originate from sewage sludge, residual plastic film, contaminated irrigation water, and atmospheric deposition (Aristizabal et al., 2024). Studies indicate that MPs can be absorbed by crops and accumulate in edible parts, with higher contamination risks observed in leafy vegetables and tuber crops (Wang et al., 2023). Aquatic products, especially filter-feeding bivalves (e.g., oysters, mussels, clams), accumulate large amounts of MPs by filtering seawater, representing an important pathway for human dietary exposure. Various polymer types of MPs have been detected in bivalve samples collected from seas such as the Mediterranean and the North Sea (Vethaak and Leslie, 2016). Food processing and packaging also pose contamination risks: MPs released from bottled water, nylon tea bags during brewing, and particles entering food from plastic equipment wear have been confirmed by research (Leslie et al., 2022; Ragusa et al., 2021). Particularly concerning is the continuous release of MPs from plastic-packaged foods during storage due to material aging and degradation; this “secondary contamination” further increases exposure risk.

### 2.3 Human exposure pathways

The main pathways for human exposure to MPs include ingestion, inhalation, and dermal contact. Among these, ingestion and inhalation are currently considered the most important exposure routes due to their high frequency of exposure, potentially significant doses, and strong support from human biomonitoring data.

Ingestion is one of the primary pathways for human exposure to MPs, mainly entering the body through contaminated food and drinking water. Seafood (e.g., fish, shellfish, crustaceans) serves as a key vector for MPs, as they ingest MPs from contaminated water and accumulate them in their digestive systems, becoming a major source of human dietary exposure. Consuming inadequately washed or uneviscerated seafood may directly lead to microplastic intake (Rochman et al., 2016). Furthermore, the widespread presence of MPs in sea salt further confirms the pervasiveness of water pollution and its transfer through the food chain (Karami et al., 2017). Drinking water is also a significant source of microplastic ingestion, with both tap water and bottled water having been detected to contain MPs, potentially originating from environmental water pollution, migration from packaging materials, or introduction during production (Koelmans et al., 2017). In agricultural systems, using water sources contaminated with MPs for irrigation, or applying sewage sludge and fertilizers containing MPs, may lead to microplastic accumulation in fruits, vegetables, and other agricultural products (Corradini et al., 2019). Processed foods (e.g., sugar, honey, beer) have also been reported to contain MPs, potentially introduced during raw material collection, processing, packaging, or storage (Li et al., 2022a; Liebezeit and Liebezeit, 2014). This evidence collectively indicates that MPs are ubiquitous in natural agricultural products and processed foods. After oral ingestion, MPs enter the digestive tract, where smaller particles (especially Nanoplastics; NPs) may penetrate the intestinal mucosal barrier, enter the systemic circulation, and migrate to other organs or tissues, potentially causing systemic health risks.

Inhalation is another key exposure pathway of growing concern. Airborne MPs primarily originate from urban dust, industrial emissions, tire and mechanical wear, shedding of synthetic textile fibers, and resuspension of particles from contaminated soil and water bodies (Allen et al., 2019; Liu and Schauer, 2021). These particles can undergo long-range transport via air currents and persist in the atmosphere, with higher concentrations especially in industrial and densely populated areas. The indoor environment plays a critical role in inhalation exposure (Salthammer, 2022). Synthetic fibers from carpets, furniture, and household items continuously release MPs during use, and limited ventilation often leads to higher indoor microplastic concentrations than outdoors (Dris et al., 2015), making residential and occupational environments high-risk settings. The deposition of inhaled MPs in the respiratory tract depends on their size and morphology: larger particles are mostly retained in the upper respiratory tract, while smaller particles like NPs can reach deep into the alveolar region and potentially cross the blood-air barrier into the circulatory system. Studies suggest such particles may be associated with lung inflammation, oxidative stress, and other respiratory diseases (Dris et al., 2015; Campen et al., 2024). The detection of MPs in human lung tissue further confirms the reality of inhalation exposure and its potential health risks, raising widespread concern about potential chronic effects from long-term exposure (Amato-Lourenço et al., 2021; Horvatits et al., 2022).

Dermal contact is generally considered a less significant exposure pathway. Although intact skin acts as an effective physical barrier against larger microplastic particles, exposure can still occur under certain conditions. MPs may enter the body through damaged skin, hair follicles, or sweat glands, or indirectly through frequent direct use of cosmetics and personal care products containing MPs (e.g., scrubs, toothpaste). Activities in contaminated freshwater or seawater environments, such as swimming, may also lead to prolonged skin contact with MPs. Although healthy skin provides a degree of protection, compromised or sensitive skin may allow smaller particles—particularly NPs—to penetrate the epidermis or interact with hair follicles (Vethaak and Leslie, 2016). Due to their extremely small size and high specific surface area, NPs exhibit greater potential for skin penetration and may induce systemic health effects by interacting with skin cells (Lai et al., 2022; Li Q et al., 2024). Currently, direct evidence for dermal absorption of MPs remains limited (Urban et al., 2000). However, occupational settings involving long-term, high-intensity exposure (e.g., plastic manufacturing, waste handling) are considered potential significant pathways. Although sufficient data confirming significant health risks from dermal contact is lacking (Abafe et al., 2024), the ongoing increase in environmental microplastic pollution underscores that this exposure pathway cannot be ignored.

## 3 Microplastics and human reproductive health

### 3.1 Impact on fertility

Given their physical and chemical properties, microplastic pollution poses a new threat to both male and female reproductive systems (Geng et al., 2023; Hong et al., 2023; Gewert et al., 2015). Studies indicate that exposure to MPs and their associated chemicals (such as phthalates and bisphenol A) may affect spermatogenesis, reduce sperm quality, and disrupt hormonal balance in males. *In vivo* experiments have demonstrated that inflammation and oxidative stress induced by MPs are associated with decreased sperm motility and viability (Hou et al., 2021). Furthermore, MPs retained in face masks may also adversely affect mammalian reproductive function, manifesting as reduced sperm motility and progressive motility. A study on bovine epididymal sperm showed that microplastic exposure could induce reactive oxygen species (ROS) generation, trigger apoptosis, and significantly decrease the blastocyst formation rate (Grechi et al., 2024). Another study revealed that after 21 days of continuous exposure to MPs, adult male C57BL/6J mice exhibited significant disruption of pathways related to spermatogenesis and oxidative stress (Lin et al., 2024).

In the female reproductive system, MPs may impair ovarian function, disrupt hormonal balance, and weaken fertility. Microplastic exposure can alter ovarian morphology and interfere with the estrous cycle, thereby affecting ovulation and embryo implantation processes (An et al., 2021). Research has found that polystyrene MPs can activate the Wnt/β-catenin signaling pathway, leading to ovarian fibrosis in rats, and induce apoptosis of ovarian granulosa cells via oxidative stress, ultimately resulting in decreased ovarian reserve (An et al., 2021). Additionally, polystyrene MPs can trigger ferroptosis in ovarian granulosa cells, thereby impairing follicular development and causing ovarian fibrosis (An et al., 2021). In female fish models, microplastic exposure led to significant bioaccumulation in the intestines and ovaries, disrupted the regulatory function of the hypothalamic-pituitary-gonadal (HPG) axis, and reduced vitellogenin levels and reproductive capacity (Li et al., 2022b). Moreover, endocrine-disrupting chemicals leaching from MPs may alter estrogen and progesterone levels, which are crucial for maintaining female reproductive health (Donoghue et al., 2017; Palioura and Diamanti-Kandarakis, 2015).

**FIGURE 2:**
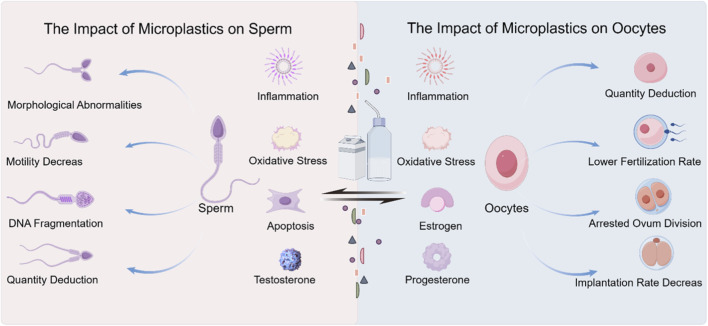
The impact of MPs on fertility.

MPs exert harmful effects on both male and female reproductive systems through multiple mechanisms, including oxidative stress, inflammation, and endocrine disruption. Regarding endocrine disruption, chemicals associated with MPs (e.g., phthalates and bisphenol A) interfere with the endocrine system by mimicking or blocking natural hormones (Chiu et al., 2020). These disruptions can affect male sperm quality, interfere with spermatogenesis, and reduce testosterone levels (Cannarella et al., 2022; Rubio et al., 2016). Endocrine disruptors can alter the balance of estrogen and progesterone in females, consequently affecting the menstrual cycle and ovarian function (Rochman et al., 2016). In terms of cellular damage, the excessive production of ROS induced by MPs can lead to cell damage in reproductive organs. Oxidative stress can affect sperm motility and DNA integrity in males (Guillante et al., 2024); in females, it may impact oocyte quality and disrupt follicular development (An et al., 2021). Regarding tissue inflammation, immune responses triggered by microplastic particles can also lead to tissue inflammation (Zhi et al., 2024). According to a previous study, persistent inflammation can damage reproductive organs, reduce fertility in both sexes, and increase the risk of reproductive system diseases (Hou et al., 2021). These mechanisms reveal the various ways MPs harm reproductive health, highlighting the urgency of addressing microplastic pollution and its associated risks (Figure 2).

### 3.2 Impact on pregnancy

MPs can cross the placental barrier, potentially exposing the developing fetus to associated risks during gestation. It has been detected in human placental tissue, raising concerns about their possible invasion of the fetal circulatory system (Ragusa et al., 2021). MPs may not only interfere with the placental functions of gas and nutrient exchange but also induce local inflammatory responses. Associated adverse pregnancy outcomes include preterm birth and fetal growth restriction (Luo et al., 2019). A clinical study further indicated that the presence of plastic particles in the placentas of young women from the general population was associated with intrauterine fetal growth retardation (Chen G. et al., 2023). It has also been confirmed that exposure of pregnant mice to MPs exerts dose-dependent adverse effects on fetal development and maternal physiology (Chen et al., 2024). Furthermore, MPs often carry two types of endocrine disruptors—phthalates and bisphenol A—which may interfere with normal fetal development and increase long-term health risks. Exposure to these chemicals during early pregnancy may impair fetal formation and development, manifesting as growth retardation (Stevens et al., 2023). Studies suggest that gestational exposure to phthalates may disrupt thyroid system function (Derakhshan et al., 2021), promote excessive weight gain, and induce insulin resistance and pancreatic β-cell dysfunction, thereby increasing the risk of gestational diabetes (Filardi et al., 2020). Although preliminary evidence exists for microplastic transfer in human placenta, current animal studies provide substantial support for their potential impacts, underscoring the urgent need for further research to fully assess their effects on human pregnancy.

**FIGURE 3:**
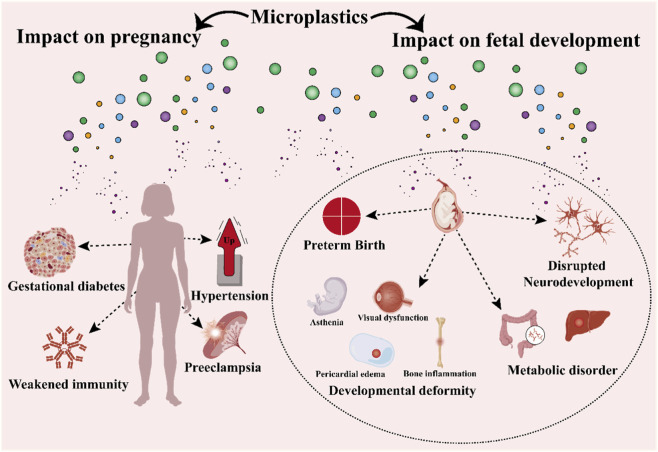
The impact of MPs on pregnancy and fetal development.

The health effects potentially arising from maternal microplastic exposure can influence pregnancy outcomes. Research suggests that maternal exposure to MPs and their associated chemicals may lead to systemic inflammation, oxidative stress, and abnormal hormone levels (Filardi et al., 2020). Conditions associated with these states, such as preeclampsia, gestational diabetes, and hypertension, pose threats to both the mother and the fetus. Additionally, MPs may impair maternal immune system function, reducing the body’s adaptive capacity during pregnancy, thereby increasing the risk of adverse outcomes such as miscarriage or preterm birth. More importantly, the accumulation of MPs in the placenta may hinder its normal function, reducing the nutrients and oxygen received by the fetus, potentially leading to growth restriction or developmental defects (Luo et al., 2019). These outcomes emphasize the urgency of understanding microplastic exposure during pregnancy and its implications for maternal-fetal health (Figure 3).

### 3.3 Impact on fetal development

Placental transfer is the primary route for MPs to reach the fetus after maternal exposure via ingestion, inhalation, or dermal contact. Both human and animal studies indicate that once MPs and their associated compounds enter the maternal bloodstream, they can cross the placental barrier into the fetal circulatory system (Teuten et al., 2009). This transfer process may disrupt normal placental function, leading to reduced oxygen and nutrient supply to the fetus during gestation. One study reported that maternal exposure to MPs could induce placental damage in mice, accompanied by metabolic disturbances and dysregulated gene expression (Wang et al., 2025). Furthermore, MPs and the endocrine disruptors they carry (e.g., phthalates and bisphenol A) may interfere with fetal development, increasing the risk of intrauterine growth restriction, preterm birth, and long-term developmental disorders. An experiment on pregnant mice confirmed that microplastic particles could induce fetal neurodevelopmental toxicity through oxidative damage and inhibition of gamma-aminobutyric acid synthesis in the brain (Yang et al., 2022). The study also observed significant fetal growth restriction following exposure to MPs during late pregnancy, with fetal weight reduced by up to 12% in the high-exposure group (Aghaei et al., 2022). The detection of MPs in amniotic fluid and fetal tissues has been associated with oxidative stress and inflammatory responses, which may further compromise pregnancy outcomes and fetal health (Luo et al., 2019). These findings underscore the critical need for further research into the fetal exposure pathways of MPs and their potential health impacts.

During critical stages of fetal development, the toxicological effects of microplastic exposure may negatively impact pregnancy outcomes. MPs and their associated chemicals (e.g., phthalates and bisphenol A) can disrupt cellular differentiation and organogenesis in early pregnancy by interfering with hormonal signaling pathways. Such interference may lead to congenital defects or organ hypoplasia (Magosso et al., 2025; Zhou et al., 2024). During the mid-to-late stages of pregnancy, microplastic-induced inflammation and oxidative stress can impair placental function, reducing the oxygen and nutrients received by the fetus, consequently leading to preterm birth or intrauterine growth restriction (Ragusa et al., 2021). Additionally, endocrine-disrupting chemicals may alter the developmental programming of the embryonic neural and endocrine systems, resulting in long-term metabolic and neurodevelopmental issues (Luo et al., 2019). These toxicological consequences highlight the necessity of reducing maternal microplastic exposure and fetal susceptibility during critical developmental windows (Figure 3).

### 3.4 Impacts on infants and children

Infants and children exhibit significantly higher susceptibility to microplastic exposure due to their immature physiological structures, unique behavioral patterns, and critical developmental stages. During this period of rapid growth, their metabolic systems, immune mechanisms, and blood-brain barrier are not fully developed, leading to reduced barrier function and clearance efficiency for environmental pollutants. Consequently, MPs and associated chemical contaminants are more likely to accumulate in the body, posing potential threats to multiple organ systems (Koelmans et al., 2020). A study on juvenile mice indicated that oral administration of MPs induced severe toxicological effects in a dose-dependent manner, including intestinal barrier damage and liver injury (Li H et al., 2024). Another study using a weaned piglet model found that dietary polystyrene MPs impaired intestinal angiogenesis via the ROS/METTL3 pathway and increased the risk of diarrhea (Zou et al., 2024). Feeding practices represent a primary pathway for microplastic exposure in infants. Heating infant formula in plastic bottles accelerates the breakdown of these materials at high temperatures, releasing substantial quantities of microplastic particles (Koelmans et al., 2020). Research suggests that millions of microplastic particles may be released per liter of formula, leading to significantly higher exposure levels per unit body weight in infants compared to adults (Mohamed Nor et al., 2021). Beyond formula feeding, MPs have also been detected in human breast milk. A study involving multiple healthy lactating women reported the presence of MPs in approximately 38.98% of breast milk samples (Saraluck et al., 2024), indicating that even breast milk, the ideal nutritional source for infants, may constitute an exposure route. An *in vitro* study simulating infant gastric digestion found that MPs in milk adversely affected the digestive process, reduced the digestibility of milk proteins, and potentially contributed to chronic health issues (Kaseke et al., 2025). Concurrently, exposure to polyethylene MPs disrupted the composition of the infant gut microbiota, increasing the alpha diversity and abundance of potential pathogens (Fournier et al., 2023). Regarding non-dietary exposure pathways, infants and young children are more vulnerable to inhaled MPs due to their shorter height, prolonged contact with floors, higher respiratory rates, and immature respiratory systems (Ragusa et al., 2021). Indoor MPs primarily originate from synthetic textiles, household dust, and the degradation of plastic products. Areas of particular concern include children’s play zones, where exposure risks are elevated (Wright and Kelly, 2017). An environmental survey in the United States indicated that the average concentration of airborne MPs in children’s play areas was approximately five times higher than in non-play areas (Koutnik et al., 2023), suggesting that children may inhale significant quantities of MPs during crawling and play activities, substantially increasing their exposure risk.

**FIGURE 4:**
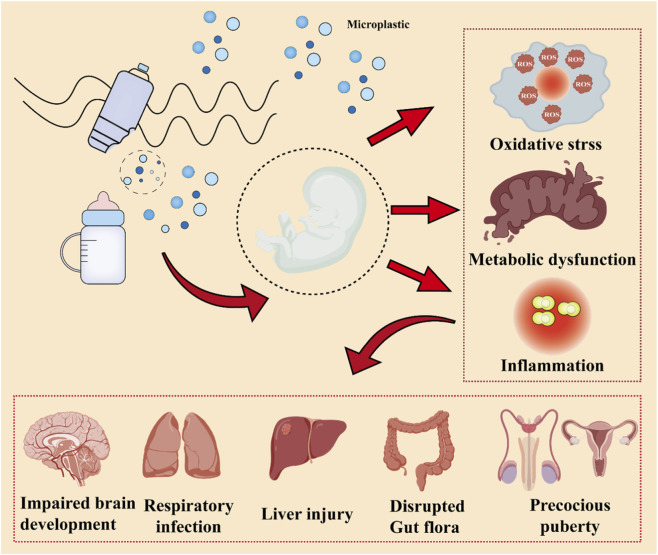
The impact of MPs on infants and young children.

Early-life exposure to MPs may lead to multi-system and multi-level health effects in infants and children, including developmental toxicity, neurotoxicity, and disruptions to endocrine and immune functions (Islam et al., 2025; Mohamed Nor et al., 2021). During infancy, the immune, endocrine, and nervous systems are in a highly dynamic state of development, making them particularly sensitive to environmental pollutants (Koelmans et al., 2020). MPs and their chemical additives (e.g., phthalates, bisphenol A) can interfere with hormonal signaling pathways, affecting critical processes such as brain development, metabolic balance, and sexual maturation (Luo et al., 2019; Ragusa et al., 2021). Exposure to MPs during critical periods of neurodevelopment may also lead to aberrant synaptic transmission, increased oxidative stress, and neuroinflammation, potentially resulting in behavioral abnormalities, impaired learning and memory, and cognitive deficits (Islam et al., 2025). Numerous studies suggest that the effects of microplastic exposure during pregnancy and early life may persist into childhood, adolescence, and even adulthood, significantly increasing the risk of neurodevelopmental disorders such as attention-deficit/hyperactivity disorder and autism spectrum disorder (Koelmans et al., 2020). Furthermore, MPs can trigger a persistent low-grade inflammatory state and immune dysregulation. They not only pose a direct threat to children’s health but may also impact the development and programming of the fetal immune system via placental transmission, increasing the likelihood of developing allergic diseases, asthma, and autoimmune disorders later in life (Figure 4).

In summary, infants and young children represent a high-risk population for microplastic exposure. The potential health risks they face necessitate heightened attention from families, healthcare professionals, and public health authorities.

## 4 Mechanisms of toxicity

### 4.1 Physical effects

The physical toxicity mechanisms of MPs primarily stem from their inherent physical attributes, such as size, shape, and surface characteristics. These attributes can trigger direct physical interactions at multiple biological levels (from cells and tissues to organs), leading to a series of adverse effects.

At the macroscopic level, physical obstruction and damage are the most direct manifestations. Studies indicate that zooplankton and filter-feeding organisms (such as certain fish and bivalves) can ingest environmental MPs, leading to particle accumulation in the digestive tract, causing physical blockage and false satiety, thereby suppressing feeding behavior. Chronic insufficient energy and nutrient intake can ultimately result in growth retardation, reduced reproductive capacity, and even individual mortality (Roman et al., 2020; Yagi et al., 2022). It is particularly noteworthy that irregularly shaped MPs (e.g., fibers or fragments), due to their sharp edges, are more likely to cause mechanical damage to the digestive tract lining. This not only exacerbates local inflammatory responses but also facilitates the intrusion of other harmful substances.

Once MPs enter an organism, they can be recognized by the immune system as “foreign particles”, triggering innate immune responses, promoting the release of pro-inflammatory cytokines, and leading to persistent inflammation. This process can further elevate oxidative stress levels, causing cellular dysfunction or even apoptosis (Zheng et al., 2023). For instance, in mammalian intestines, long-term microplastic exposure is closely associated with an increased risk of inflammatory bowel disease (Ji et al., 2023). Simultaneously, MPs can disrupt the composition and diversity of the gut microbiota, reducing beneficial bacteria and increasing potential pathogens, thereby disturbing microecological balance (Li et al., 2020; Zou et al., 2023).

At a more microscopic level, NPs (size <100 nm), due to their scale being comparable to biomacromolecules, exhibit strong membrane penetration capabilities. Their hydrophobic surfaces can directly embed into the phospholipid bilayer of cell membranes, disrupting structural integrity, increasing membrane permeability, inducing intracellular ion imbalance and content leakage, and facilitating the entry of exogenous pollutants into cells (Wang et al., 2024). Furthermore, NPs can enter the circulatory system, causing embolisms in microvessels, obstructing blood flow, and leading to local tissue ischemia and necrosis (Mallapaty, 2025). Studies have also found that such particles can cross biological protective barriers like the blood-brain barrier and placental barrier, accumulate in fetal tissues, and disrupt the maternal-fetal interface microenvironment through a combination of physical occupation and inflammation induction, potentially interfering with embryonic development (Yu et al., 2024). While existing research often focuses on their role as carriers of additives, it is widely acknowledged that microplastics themselves “can directly cause physical damage and metabolic disorders in organisms” (Koelmans et al., 2022; Seeley et al., 2023).

### 4.2 Chemical effects

Microplastics pose ecotoxicological risks through two primary, distinct pathways: the direct physical effects of the particles, and chemical-mediated effects from the release of manufacturing additives (Xu et al., 2025). Accurately assessing their environmental and health impacts requires considering this fundamental distinction. The chemical-mediated toxicity is largely driven by the leaching of additives. Recent molecular dynamics simulations have elucidated the release kinetics of common additives like BPA, DBP, and DEHP from polymers such as polyethylene and polyvinyl chloride (Xu et al., 2025). The release of additives mainly relies on their diffusion through cavities within the polymer matrix, with internal diffusion within the particle serving as the rate-limiting step. The release rate is regulated by a combination of factors: shorter polymer chains (e.g., PE10, PVC10) or smaller microplastic particle sizes reduce the encapsulation of additives and weaken the Lennard-Jones interaction energy between the polymer and additives, thereby accelerating their migration to the surface; conversely, longer chains and larger particles inhibit release. The molecular characteristics of the additives themselves are also critical: BPA, with its smaller molecular radius and volume, encounters lower steric hindrance within the matrix (Urban et al., 2000; Horvatits et al., 2022), leading to easier and faster release, whereas larger molecules like DEHP face greater diffusion resistance. Environmental conditions similarly exert significant influence on release behavior: elevated temperatures (e.g., 320K, 350K) can relax the polymer network by enhancing the thermal motion of polymer chains and additive molecules, markedly accelerating release; increased salinity (1 mol/L NaCl) exerts a more complex effect—while salt ions may increase chain spacing through charge shielding and electrostatic interactions, potentially facilitating internal diffusion, experimental evidence suggests that salinity may ultimately suppress the overall desorption of additives into water (Dhavamani et al., 2022). Molecular dynamics analysis of additive center-of-mass trajectories further confirms that additive molecules overcome energy barriers between cavities through thermal fluctuations, migrating stepwise to the particle surface in a “hopping” manner.

These molecular-scale insights are crucial, as they systematically elucidate the regulatory mechanisms of additive release. They quantitatively reveal how polymer characteristics, additive properties, and environmental conditions jointly determine diffusion and release kinetics (Xu et al., 2025). This refinement of the theoretical framework concerning the dual risks of microplastics (Barrick et al., 2021) underscores a critical point: the release kinetics of additives are a pivotal precursor step governing ecological exposure and subsequent toxicity. Consequently, the overall ecotoxicological risk of MPs arises not merely from their physical presence, but from the dynamic interaction between those inherent physical hazards and the chemical hazards unleashed by the leaching of additive mixtures—a process itself governed by a complex interplay of materials science and environmental parameters (Barrick et al., 2021; Campen et al., 2024).

Building upon this understanding that chemical hazard is often mediated by released or releasable agents, MPs themselves are largely chemically inert. Their significant chemical toxicity in practice stems from two principal, and often co-occurring, aspects: their role as vectors for various environmental chemicals, and the release of additives incorporated during manufacturing within organisms (Boháčková and Cajthaml, 2024).

MPs can act as vectors, adsorbing environmental pollutants such as heavy metals (e.g., lead, cadmium) and persistent organic pollutants (e.g., polychlorinated biphenyls, polycyclic aromatic hydrocarbons) (Boháčková and Cajthaml, 2024). These pollutants can desorb from the microplastic particles or enter the organism along with the particles, interfering with normal physiological functions. For example, heavy metals adsorbed onto MPs can disrupt enzyme activity and cellular function, leading to DNA damage and oxidative stress; whereas persistent organic pollutants are closely associated with developmental toxicity, immunosuppression, and endocrine disruption (Gao et al., 2021).

Additives used in plastic production are also a significant source of toxicity. Research shows that phthalates, commonly used as plasticizers, exhibit endocrine-disrupting effects by interfering with hormonal signaling pathways (Abafe et al., 2024). Gestational exposure to phthalates may disrupt the balance of pregnancy-related hormones like progesterone and estrogen, increasing the risk of preeclampsia, gestational diabetes, and preterm birth. Animal studies further confirm that phthalate exposure is associated with prenatal growth restriction and impaired placental development (Martínez-Razo et al., 2021). Another common additive, bisphenol A (BPA), exhibits estrogen-like effects and may interfere with the natural hormonal balance during pregnancy. BPA exposure is linked to an increased risk of miscarriage, abnormal fetal development, and long-term metabolic disorders in offspring.

Moreover, when organisms are simultaneously exposed to additives carried by MPs and adsorbed pollutants, synergistic toxic effects may occur. For instance, heavy metal-induced oxidative stress might exacerbate the endocrine disruption caused by phthalates and BPA, further impairing maternal health and fetal development (Borriello et al., 2025; Carter and Blizard, 2016). Such cumulative toxicant load may elevate the risk of adverse pregnancy outcomes, including preterm birth, fetal growth restriction, and increased susceptibility to chronic diseases in offspring.

### 4.3 Synergistic interactions

The environmental risks posed by MPs extend far beyond the simple sum of their individual physical or chemical effects, primarily stemming from complex interactions and synergistic mechanisms among multiple factors. This synergy enables MPs to act as efficient vectors and toxicity amplifiers for environmental pollutants, posing potential systemic threats to ecosystem function and human health.

At the toxicological mechanism level, significant mutual enhancement exists between physical and chemical effects. In their role as physical vectors, MPs, by virtue of their large specific surface area and strong hydrophobicity, can adsorb and transport various harmful pollutants (such as persistent organic pollutants and heavy metals) directly into organisms. This process not only bypasses the organism’s external physical barriers but also creates localized pollution hotspots (e.g., in the intestines or gills) where pollutant concentrations far exceed background environmental levels, substantially increasing the actual exposure dose. More critically, MPs can facilitate the transport and redistribution of pollutants within the organism. For instance, after crossing the intestinal mucosa and entering the lymphatic or circulatory systems, MPs may act as a “Trojan horse,” delivering toxic substances to metabolically active and sensitive tissues and organs, thereby significantly expanding their scope and severity of harm (Hu et al., 2022).

Conversely, chemical toxicity can exacerbate physical damage induced by MPs. Additives inherent to MPs and adsorbed exogenous pollutants often exhibit significant cytotoxicity, inducing strong oxidative stress and inflammatory responses within organisms. Such chemical stressors persistently deplete cellular antioxidant capacity and impair tissue self-repair mechanisms, hindering the effective healing of physical damage caused by MPs (such as mucosal abrasion and membrane structural damage). This ultimately traps tissues in a vicious cycle of “damage–inflammation–impaired repair.”

Beyond physicochemical synergy, environmental stress factors can also significantly amplify the overall ecological risk of MPs. Factors induced by climate change, such as rising temperatures, altered precipitation patterns, and increased frequency of extreme weather events, directly influence the environmental behavior and fate of MPs. For example, higher temperatures can accelerate the photo-aging and mechanical fragmentation of plastic products, increasing the generation of secondary microplastic particles and raising the likelihood of ingestion or inhalation by organisms (Ding et al., 2020). Under combined stress scenarios involving heat stress, water scarcity, and co-exposure to other pollutants, the effects of MPs and climate change may further exacerbate the vulnerability of certain biological populations. Studies indicate that pregnant organisms simultaneously exposed to MPs and heat stress experience a significantly elevated physiological load. Furthermore, climate-related disasters such as floods and wildfires can compromise water and food safety, promote the dispersion of microplastic pollution, and thereby pose combined threats to vulnerable groups like pregnant women and infants (Sarasamma et al., 2020).

### 4.4 Cell-type-specific toxicity

The toxic effects of microplastics exhibit significant heterogeneity across different cell types, and this specificity of interaction underpins their ability to cause target organ damage. Within the reproductive system, trophoblasts are central to placental function during pregnancy. Research indicates that polystyrene nanoplastics (PS-NPs) can be internalized by these cells and accumulate in mitochondrial regions, inducing a drop in mitochondrial membrane potential and a burst of reactive oxygen species (Basini et al., 2021). This activates the mitochondrial apoptosis pathway, characterized by downregulation of Bcl-2 and upregulation of Cleaved-caspase-3, ultimately leading to excessive apoptosis, providing a direct cytological mechanism for the increased miscarriage risk associated with MPs exposure (Wan et al., 2024). In the ovary, granulosa cells are vital for follicular development. Exposure to PS-NPs can disrupt the redox homeostasis of porcine ovarian granulosa cells, stimulating superoxide anion production and impairing their steroid hormone (e.g., estradiol and progesterone) synthesis and secretion functions (Basini et al., 2021); similarly, in a mussel model, aged PET micro/nanoplastics induced lysosomal damage and early mortality in granulosa cell subsets, accompanied by tissue-level degenerative changes (Hara et al., 2025). Within the nervous system, nanoplastics capable of crossing the blood-brain barrier can be taken up by neurons and microglia. Following neuronal uptake, MPs trigger apoptosis or ferroptosis by inducing excessive ROS production, lipid peroxidation, and mitochondrial dysfunction, leading to hippocampal neuron loss associated with cognitive decline (Chen et al., 2025; Jin et al., 2022). Concurrently, activated microglia release pro-inflammatory factors such as TNF-α, exacerbating the neuroinflammatory milieu (Lee et al., 2022). In the immune system, macrophages, as the primary clearing cells, experience lysosomal damage and lipid accumulation after phagocytosing MPs (Florance et al., 2022). This is followed by activation of the MAPK/NF-κB pathway and the NLRP3 inflammasome, resulting in increased ROS (Zhu et al., 2017; Tang et al., 2022; Yin et al., 2023), release of pro-inflammatory cytokines, and eventual apoptosis (Busch et al., 2022; Deng et al., 2017; Dan et al., 2025; Tang et al., 2022). Regarding adaptive immunity, PS-NPs can suppress the expression of activation markers on T cells and the secretion of effector molecules like IL-2 and IFN-γ, weakening the cellular immune response (Li et al., 2022c). Furthermore, long-term exposure may disrupt T helper cell balance (e.g., favoring a Th2 bias) and affect antibody class switching in B cells, compromising humoral immune function (Kusma et al., 2024). These cell-type-specific patterns of damage collectively map the precise pathways through which MPs drive cellular dysfunction towards organ pathology.

## 5 Mitochondrial dysfunction and accelerated aging

Following the discussion on the detection and potential impacts of MPs in the female placenta, understanding how these pollutants accelerate the aging process has become a critical issue (Balistreri et al., 2024). The global trend of population aging is intensifying, and aging itself is a pathophysiological process characterized by progressive and irreversible decline in organ function, significantly increasing susceptibility to age-related diseases such as Alzheimer’s disease and atherosclerosis (Della Torre et al., 2024; Kloet et al., 2015; Balistreri et al., 2024). Recent studies indicate a clear association between microplastic exposure and both aging and the development of various age-related diseases. For instance, clinical research has found that patients with MPs detected in carotid artery plaques have a significantly higher risk of myocardial infarction, stroke, or all-cause mortality (Marfella et al., 2024). Animal experiments have also confirmed that exposure to MPs can induce tissue aging and functional decline in multiple organs, including the heart, kidneys, skin, and brain (Wang et al., 2024b; Pan et al., 2024; Han et al., 2024; Zhou et al., 2023). These observations suggest that MPs may systematically accelerate organismal aging through common cellular and molecular mechanisms.

The process begins with cellular internalization. Microplastics (MPs) and nanoplastics (NPs) enter cells through various active and passive endocytic pathways, with efficiency dependent on physicochemical properties such as size, surface chemistry, and charge (Hesler et al., 2019; Lee et al., 2021). Smaller NPs primarily utilize clathrin- or caveolin-mediated endocytosis, while larger MPs/NPs are mainly internalized via macropinocytosis (Kong et al., 2025; Liu et al., 2021). Particles can also cross physiological barriers; for example, small polystyrene NPs traverse the placental barrier in *ex vivo* perfusion models (Grafmueller et al., 2015; Ragusa et al., 2021). Furthermore, MP/NP exposure itself may compromise membrane integrity, facilitating passive entry (Lu et al., 2022). This internalization represents the first step in MP/NP-induced cellular damage.

One of the core mechanisms by which MPs accelerate aging is the induction of mitochondrial dysfunction. Mitochondria, the “powerhouses” of the cell, experience functional decline with age, manifested as the accumulation of mitochondrial DNA (mtDNA) mutations, dysregulation of quality control systems, and impaired biogenesis, which is considered a hallmark of aging (Martini and Passos, 2023). Studies have confirmed that MPs can enter cells via pathways such as macropinocytosis, clathrin- or caveolin-mediated endocytosis (Khan and Jia, 2023; Liu et al., 2021), subsequently targeting and damaging mitochondria, leading to functional abnormalities such as decreased mitochondrial membrane potential and impaired adenosine triphosphate (ATP) production (Li et al., 2022c). This mitochondrial damage serves as the starting point for a series of downstream pro-aging cascades.

Once internalized, MPs/NPs directly interact with mitochondria. In skin cells like HaCaT and JB6-C30, particles localize near mitochondria in a time- and dose-dependent manner, inducing mitochondrial oxidative stress and membrane depolarization (Han et al., 2024). This leads to a significant increase in mitochondrial reactive oxygen species (mtROS) production, creating a critical oxidative environment (Zhao et al., 2024; Zhou et al., 2023).

Specifically, MPs drive the aging process by disrupting mitochondrial function, primarily involving the following three interconnected pathways:

First, microplastic-induced mitochondrial damage leads to excessive generation of reactive ROS. Under physiological conditions, mitochondria produce ATP via oxidative phosphorylation while leaking a small number of electrons to form ROS. In aging cells, an imbalance in antioxidant defense mechanisms leads to ROS accumulation (Binatti et al., 2021; Maharana et al., 2026). Microplastic exposure exacerbates this process; for example, in cardiomyocytes, MPs induce a burst of mitochondrial ROS (mtROS), subsequently activating the expression of aging-related signaling molecules such as p53, p21, and p16, promoting cellular senescence. The use of ROS scavengers can effectively mitigate this process (Wang et al., 2024b; Yang Q. et al., 2023). Excessive ROS can also activate inflammatory signaling pathways such as NF-κB/NLRP3, TGF-β/Smad2/3, and p38 MAPK, creating a vicious cycle between oxidative stress and chronic inflammation (He et al., 2022; Wang et al., 2023).

The molecular pathway involves specific signaling activation: the excessive mtROS contributes to DNA damage and activates stress-responsive kinases including p38 MAPK. These signals converge to upregulate the core senescence regulators p53, p21, and p16 through well-defined transcriptional mechanisms (Han et al., 2024).

Second, mtDNA released from damaged mitochondria is a key mediator triggering chronic inflammation. Due to the lack of histone protection and proximity to the electron transport chain, mtDNA is highly susceptible to ROS attack and oxidative damage, thereby possessing stronger pro-inflammatory potential (Zhao et al., 2021). MPs can induce the activation of mitochondrial membrane channel proteins (e.g., VDAC) or promote the aggregation of gasdermin D (GSDMD) on mitochondria, leading to the release of mtDNA into the cytoplasm (Han et al., 2024). The release mechanism specifically involves GSDMD pore formation on mitochondrial membranes, which facilitates mtDNA release distinct from mitochondrial permeability transition pore-mediated pathways (Han et al., 2024; Wang et al., 2024). Cytosolic mtDNA is then sensed by cGAS, activating the cGAS-STING-NF-κB signaling axis, or directly activating the AIM2 inflammasome, thereby driving the massive expression of pro-inflammatory factors such as interleukins, initiating and maintaining a state of chronic inflammation, and accelerating tissue aging (Shen et al., 2022; Wang et al., 2024b; Chung et al., 2009). This mechanism corroborates the AIM2 inflammasome activation pathway observed in our skin cell model (Cui et al., 2024).

Upon entering the cytosol, mtDNA binds to cyclic GMP-AMP synthase (cGAS), catalyzing 2′3′-cGAMP synthesis that activates STING. This triggers TBK1 phosphorylation and subsequent IRF3/NF-κB activation, leading to type I interferon and pro-inflammatory cytokine production (Shen et al., 2022). Simultaneously, mtDNA activates the AIM2 inflammasome through direct DNA binding, resulting in caspase-1 activation and IL-1β/IL-18 maturation (Cui et al., 2024).

Third, MPs interfere with mitochondrial quality control mechanisms, particularly mitophagy and mitochondrial biogenesis. Mitophagy is responsible for clearing damaged mitochondria and is crucial for maintaining a healthy mitochondrial network, preventing mtDNA leakage, and excessive ROS production (Sliter et al., 2018). However, microplastic exposure can disrupt this process: on one hand, long-term exposure to PS-MPs inhibits mitophagy in mouse testicular tissue, leading to abnormal accumulation of damaged mitochondria and mtROS, promoting tissue aging (Wu et al., 2023); on the other hand, MPs may also induce abnormal mitophagy by overactivating the AMPK-ULK1 signaling pathway, exacerbating neuronal damage (Huang et al., 2023). Additionally, MP/NP exposure can disrupt mitochondrial dynamics, inducing excessive fission that further compromises mitochondrial function and cellular homeostasis (Zhao et al., 2024). Simultaneously, MPs can also inhibit the AMPK-PGC-1α signaling pathway, downregulating the expression of the key regulator of mitochondrial biogenesis, PGC-1α, hindering the generation of new functional mitochondria, thereby weakening cellular repair and renewal capacity and exacerbating aging-related damage (Zhang et al., 2022; Li et al., 2021). This occurs through mitochondrial stress-induced inhibition of AMPK activation, leading to reduced phosphorylation and transcriptional activity of PGC-1α, ultimately suppressing mitochondrial biogenesis (Zhao et al., 2024; Chung et al., 2009).

**FIGURE 5:**
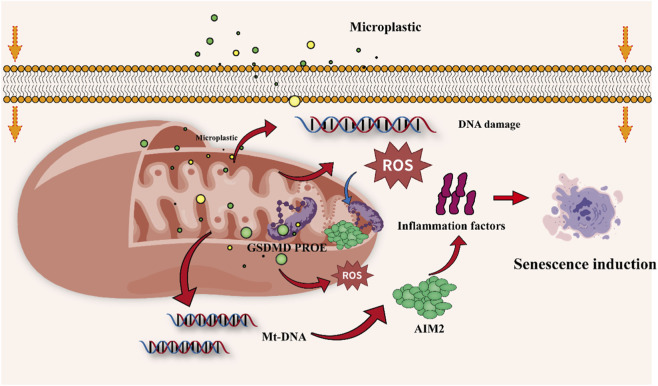
Mechanistic pathway of microplastic-induced cellular senescence and necrosis via mitochondrial dysfunction.

In summary, MPs induce mitochondrial dysfunction through direct or indirect pathways, subsequently triggering excessive ROS accumulation, mtDNA-mediated chronic inflammation, and imbalance in mitochondrial quality control. Together, these constitute the core pathological mechanism by which MPs accelerate organismal aging and the development of age-related diseases in multiple organs (Djouina et al., 2022; Zhang et al., 2023). These mechanisms have been confirmed in various tissues such as the heart, brain, and skin, forming a coherent pathological framework from organelle damage to systemic aging. Given the central role of mitochondria in aging, these pathways represent critical targets for future research aimed at mitigating microplastic-related health hazards (Figure 5).

## 6 Research directions and preventive measures

### 6.1 Current research limitations

Despite increasing attention to microplastic pollution and its impacts, research in this field still faces significant limitations, particularly the lack of standardized methods for studying the health and environmental effects of MPs. These methodological inconsistencies hinder the ability to obtain reliable, reproducible, and comparable results, limiting a comprehensive understanding of microplastic impacts.

The absence of a unified scheme for describing and classifying MPs is a major obstacle. Due to the vast heterogeneity of MPs in size, shape, chemical composition, and origin (Koelmans et al., 2020), standardizing analytical methods for their detection and characterization is challenging. For instance, different studies employ varying size thresholds to define MPs; some include particles as large as 5 mm, while others consider only particles smaller than 1 mm. Such discrepancies make it difficult to compare different studies and integrate data to draw meaningful conclusions. The lack of clear protocols for sampling MPs from environmental media—including soil, water, air, and food—is another issue. Different studies use various procedures, from filtration and sieving to complex spectroscopic methods, for isolating and measuring MPs. The use of diverse detection techniques (e.g., Scanning Electron Microscopy, Raman spectroscopy, Fourier-Transform Infrared spectroscopy) further introduces variability in sensitivity and detection limits. Owing to this inconsistency, coupled with reported variations in concentrations and distributions across different habitats, accurately determining the global prevalence of MPs remains difficult.

Methodological shortcomings also plague toxicological studies on MPs. In experimental designs, researchers often use MPs of differing sizes, shapes, and chemical compositions, along with varying exposure durations and concentrations. These variations lead to conflicting findings regarding toxicity mechanisms and dose-response relationships. For example, some studies highlight oxidative stress and inflammation as primary adverse effects, while others focus on endocrine disruption or neurotoxicity (Islam et al., 2025). The use of artificially synthesized MPs in laboratory settings introduces further uncertainty, as these particles may not accurately mimic those found in the environment. Studies often focus on a single exposure route (e.g., ingestion or inhalation), overlooking the cumulative effects of multiple pathways. Furthermore, understanding of how MPs interact with other environmental pollutants is scarce. Simulating real-world exposure scenarios in the lab—where humans are exposed to mixtures of MPs, heavy metals, and organic pollutants simultaneously—is challenging (Ragusa et al., 2021). This gap limits the practical relevance of current findings for human health and the environment.

Another significant controversy lies in the technical detection and standardization of microplastics in environmental and biological samples. The lack of universally accepted protocols for sampling, extraction, and identification leads to substantial variability in reported concentrations and types of MPs across studies, complicating exposure assessment and risk comparison. For instance, discrepancies in detection limits and specificity between spectroscopic techniques (e.g., FTIR vs. Raman) and mass spectrometry methods can result in underestimation or overestimation of particle counts (Ivleva, 2021). Furthermore, the challenge of distinguishing synthetic MPs from natural organic particles and the influence of sample matrix interference remain unresolved, raising questions about the accuracy and reliability of current detection approaches (Koelmans et al., 2020; Santos et al., 2025). Addressing these methodological controversies is essential for establishing robust exposure–response relationships and credible health risk assessments.

Beyond these methodological and experimental challenges, a more fundamental limitation arises when attempting to translate findings from model systems to human risk assessment: the uncertainty of interspecies extrapolation (He and Yin, 2023; Yee et al., 2021). This uncertainty stems from inherent differences in physiology, metabolism, exposure scenarios, and overall toxicodynamic sensitivity across species. For instance, fundamental distinctions in reproductive anatomy, hormonal regulation, and gestation between rodents and humans necessitate extreme caution when extrapolating observed effects—such as micro/nanoplastic (MNP)-induced ovarian reserve decline or placental dysfunction—to human females (Song et al., 2023; Zurub et al., 2024). While animal models are indispensable for revealing potential mechanisms (e.g., oxidative stress, endocrine disruption) and providing early hazard identification (Geng et al., 2023; Leslie and Depledge, 2020), they cannot replicate the complex reality of chronic, low-dose, multi-pollutant exposure in humans, nor can they capture human-specific genetics, behaviors, or life stages (e.g., extended reproductive lifespan) (Afreen et al., 2023; Yee et al., 2021) Consequently, quantifying the absolute risk of MPs to human reproductive health based solely on current animal data remains a significant challenge.

### 6.2 Priority areas for future research

As understanding of microplastic pollution deepens, identifying priority research areas is crucial for advancing knowledge and mitigating potential risks. Concentrating efforts on human epidemiological studies, research into microplastic toxicity mechanisms, and policy development will bridge key knowledge gaps and support informed decision-making.

Currently, there is a scarcity of epidemiological studies investigating the impact of microplastic exposure on human health. There is a pressing need for longitudinal cohort studies to assess the relationship between chronic microplastic exposure and health outcomes, such as reproductive disorders, immune dysfunction, and developmental delays in children. Studying populations with higher exposure risks is critical. Accurately measuring microplastic concentrations in human biological samples (e.g., blood, urine, tissue biopsies) is essential for linking exposure to adverse health effects.

Although evidence suggests that MPs can induce oxidative stress, inflammation, and endocrine disruption, the underlying mechanisms remain poorly understood. Investigating how MPs and associated chemicals (e.g., BPA, phthalates) interfere with hormonal signaling pathways is crucial for understanding their reproductive and developmental impacts. Studying how MPs cross biological barriers (e.g., the placenta) can provide insights into fetal developmental risks (Ragusa et al., 2021; Gautam et al., 2022). To directly bridge the translational gap from experimental models to human risk assessment, future research must strategically prioritize two complementary approaches. First, advancing human biomonitoring studies is imperative to establish direct epidemiological links between internal microplastic burden and adverse health outcomes (Liu et al., 2023; Zurub et al., 2024). Second, there is a critical need to develop and validate more human-relevant *in vitro* models, such as organoids and microphysiological systems (e.g., “tissue chips”) that accurately recapitulate human tissue physiology and disease processes (Yan et al., 2023; Young et al., 2017). These efforts will provide a more reliable and ethical foundation for risk assessment. MPs often co-exist with other pollutants (e.g., heavy metals, persistent organic pollutants). Research exploring combined toxicity is essential for assessing real-world exposure risks (Koelmans et al., 2020).

## 7 Conclusion

**FIGURE 6:**
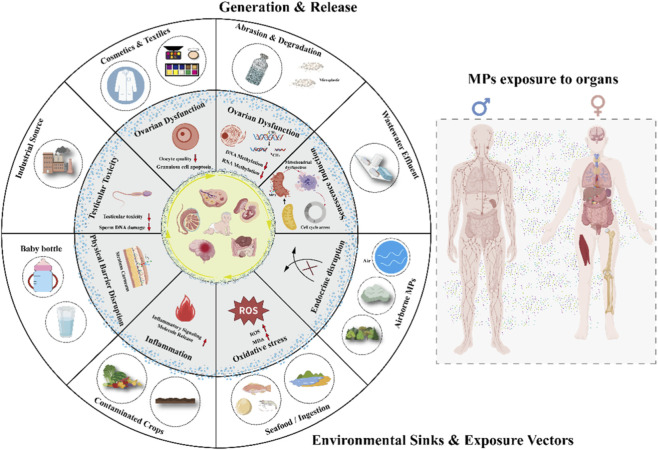
The multifaceted threat of MPs to human health across the life course.

Microplastic pollution has emerged as a severe global challenge, posing profound implications for the environment and human health. This review has highlighted the ubiquity of MPs in water, air, soil, and food systems, and identified significant human exposure pathways, primarily through ingestion, inhalation, and dermal absorption. The health hazards of MPs, particularly their impacts on reproductive health, pregnancy, and child development, are increasingly evident, with underlying mechanisms involving oxidative stress, inflammation, endocrine disruption, and placental transfer. The particular vulnerability of infants, young children, and adolescents underscores the necessity for urgent action. The physical (e.g., size, shape, density) and chemical (e.g., additives, adsorbed pollutants, leachates) properties of MPs further exacerbate their toxicity. They act as vectors for harmful substances, potentially amplifying their health effects. Furthermore, MPs interact with other environmental stressors, creating synergistic consequences that are not yet fully understood. While highly valuable, current research faces significant limitations, including a lack of standardized methodologies and insufficient long-term epidemiological data (Figure 6).
